# Investigating clinical characteristics and prognostic factors in patients with chronic osteomyelitis of humerus

**DOI:** 10.1186/s41038-019-0173-0

**Published:** 2019-12-05

**Authors:** Hongri Wu, Shengpeng Yu, Jingshu Fu, Dong Sun, Shulin Wang, Zhao Xie, Yungui Wang

**Affiliations:** 1 Department of Social Medicine and Health Service Management, Third Military Medical University, 400038 Chongqing, People’s Republic of China; 2 Department of Orthopaedics, Southwest Hospital, Third Military Medical University, Chongqing, 400038 People’s Republic of China

**Keywords:** Humeral, Osteomyelitis, Bone infection, Antibiotic cement

## Abstract

**Background:**

Chronic osteomyelitis in the humerus, which has complex neuroanatomy and a good soft tissue envelope, represents a unique clinical challenge. However, there are relatively few related studies in the literature. This article retrospectively reviewed a large case series with the aims of sharing our management experiences and further determining factors associated with the outcomes.

**Methods:**

Twenty-eight consecutive adult patients with a mean age of 36 years were identified by reviewing the osteomyelitis database of our clinic centre. The database was used to prospectively identify all osteomyelitis cases between 2013 and 2017, and all data then was retrospectively analysed.

**Results:**

The mean follow-up period was 35 months (range 24–60). The aetiology was trauma in 43% (12) of the patients and haematogenous in 57% (16) of the patients, and *Staphylococcus aureus* was a solitary agent in 50% (14) of the patients. Host-type (Cierny’s classification) was IA in 8, IIIB in 11 and IVB in 9 patients. All patients required debridement followed by the placement of a temporary antibiotic-impregnated cement spacer (rod). Seventeen patients received a cement-coated plate for internal fixation after debridement, and 13 patients needed bone grafts when the spacer was staged removed. All patients attained an infection-free bone healing state at the final follow-up. The final average DASH (disabilities of the arm, shoulder and hand) score was 18.14 ± 5.39, while 6 patients (two developed traumatic olecranarthritis, four developed radial nerve injuries) showed the lowest levels of limb function (*p* = 0.000) and were unemployed. Three patients (type I; significant difference between type I versus type III and type IV patients, *p* < 0.05) experienced recurrence after debridement and underwent a second revision, which was not related to the bone graft (*p* = 0.226) or plate fixation (*p* = 0.050).

**Conclusions:**

Humeral chronic osteomyelitis can be treated with general surgery and anti-infective therapy; medullary (type I) infection presents a challenge, and the antibiotic-coated cement plate provides favourable fixation without increasing recurrence of infections. Clinicians should be aware of potential iatrogenic nerve injuries when treating these patients with complicated cases, and an experienced surgeon may improve the outcome.

## Background

Currently, humeral osteomyelitis is relatively rare compared with the incidence of osteomyelitis of the lower limbs. The incidence of humeral osteomyelitis has been reported to compose only 2.6–13.3% of all osteomyelitis cases [1, 2], but the humerus is the most commonly affected bone in the upper limbs. The management strategies for humeral osteomyelitis mainly originate from those of osteomyelitis in the lower limbs, and of these strategies, radical debridement, delayed bone defect repair and long-term anti-infective therapy are well acknowledged [3–5]. However, resolving infections and restoring functional integrity are still challenges in the treatment of osteomyelitis. The long course of infections and repeated surgeries often lead to severe comorbidities or persistent deficits. When dealing with humeral osteomyelitis, the surrounding neuroanatomy (radial nerve) of the humerus further complicates the treatment, may be easily injured and may affect the treatment outcome [6]. Moreover, the good soft tissue envelope and good blood supply of the humerus may allow some deviations from the general management principles.

Despite the clinical challenges and characteristics, however, information on patients and treatments of humeral osteomyelitis is still limited in the literature. Street et al. [6] reported the largest case series (49) of humeral osteomyelitis to date, but the patients were all paediatric. The other reports were only case reports [7, 8] or small case series [9–11]. We retrospectively reviewed a large adult case series of humeral osteomyelitis, and we shared our experiences in treating patients and further investigated factors associated with the outcomes. Because of the unique characteristics of the humerus, we hypothesized that both the epidemiology and the treatments would be slightly different and that the factors associated with the outcomes would reflect these related variables. This series will add new informational data to the literature and improve the treatment of humeral osteomyelitis.

## Methods

We performed a 5-year cohort study by reviewing the osteomyelitis database at our tertiary clinical centre, and all adult patients who were treated for humeral osteomyelitis between 2013 and 2017 were included. Patients with humeral osteomyelitis with a duration of < 6 weeks and multifocal osteomyelitis were excluded. We identified 28 patients from all databases, which included 812 consecutive cases. The institutional review board approved this retrospective investigation, and it was conducted in conformity with the ethical principles of research.

The epidemiological characteristics, including sex, age, location, symptoms, infection duration, aetiology, Cierny-Mader classification [12], comorbidities and pathogenic organisms, were retrospectively reviewed. Treatment information in terms of antibiotic therapy and surgical procedure was investigated. Outcomes or follow-up data including the infection cure, bone healing information, post-operative disabilities of the arm, shoulder and hand (DASH) score, final occupational state and complications were prospectively recorded in the database, and these data were also retrospectively analysed. Infection cure was defined as the resolution of the clinical signs of infection and the normalization of ESR (erythrocyte sedimentation rate) and CRP (C-reactive protein). The DASH [9] score is a self-reported score. Patients attribute scores of 1 to 5 on 30 items relating to functional activities and symptoms. The raw score is then transformed to a 0 to 100 scale, whereby 0 reflects the minimum and 100 the maximum level of disability (DASH items are shown in Additional file 1).

We further explored outcome-related factors, including epidemiology (sex, age, infection duration, aetiology, comorbidity, Cierny-Mader classification) and treatments (post-debridement fixation and staged bone graft). These variables were divided into different subgroups for comparison. Numerical variables were reported as the mean ± standard deviation (SD) or median (range), and categorical variables were reported as percentages. Qualitative data were compared using the Chi-square test. An independent *t* test or a non-parametric test (Kruskal-Wallis *H* test) was used to analyse the numerical variables according to the normal distribution. The difference between the groups was considered to be significant when *p* < 0.05 in a two-sided test. Data were analysed using the SPSS 13.0 statistical software package (SPSS Inc., Chicago IL, USA).

## Results

Twenty-eight (3.4%) adult patients with humeral chronic osteomyelitis were identified from all databases, which included 812 consecutive osteomyelitis cases. The mean follow-up time was 35 months (range 24–60), and no patients were lost to follow-up (Table 1).

**Table 1:** Patient characteristics

Patients	*n*=28
Gender	
Male	16 (57.1%)
Female	12 (42.9%)
Age ( *y* )	36 (19- 62)
Infection site	
Proximal	8 (28.6%)
Middle	12 (42.8%)
Distal	8 (28.6%)
Duration of infection (*m*)	90 (2- 492)
Surgeries before	
≥One time	21 (75.0%)
None	7 (25.0%)
Aetiology	
Post-traumatic	12 (42.8%)
Haematogenous	16 (57.2%)
Organisms	
Staph.aureus	14 (50.0%)
MRSA	6 (21.4%)
None	8 (28.6%)
Cieney-mayder classification	
I A	8 (28.6%)
III B	11 (39.3%)
IV B	9 (32.1%)
Comorbidity	
Traumatic olecranarthritis	2 (7.1%)
Radial nerve injuries	4 (14.3%)
Systemic antibiotics (*w*)	4
Local antibiotics (*w*)	13 (9-26)
Extra revision	3 (10.7%)
Infection-free bone healing	28 (100%)
Follow-up (*m*)	35 (24-60)

Data are presented as n(%) or Median (range)

*MRSA* methicillin-resistant *Staphylococcus aureus*, *y* years, *n* number of patients, *w* weeks, *m* months

### Epidemiological

There were 16 males and 12 females, with a mean age of 36 years (range 19–62). The right humerus was involved in 10 patients, and the left was involved in 18 patients. The infection involved the proximal humerus in 8 patients, the shaft in 12 patients, and the distal humerus in 8 patients. Persistent pain or sinus tract was found in most patients. The infection duration ranged from 2 to 492 months. The aetiology was trauma in 43% (12) of the patients and haematogenous in 57% (16) of the patients, and 75% (21) of the patients previously sustained at least one surgery and had a recurrent illness when they were referred to our clinical centre (Table 1).

Eight patients had type IA, medullary involvement and normal host conditions. Another 11 patients and 9 patients had localized (type IIIB) and diffused (type IVB) forms, respectively, with local compromise. *Staphylococcus aureus* (*S. aureus*), a solitary agent, was found in 14 (50%) patients: 6 patients had growth of methicillin-resistant *S. aureus* (MRSA), and the cultures were negative in 8 (28%) patients. Comorbidities were common (22%): Two patients developed traumatic olecranarthritis, and their self-reported pain scores (VAS) were 5 and 4; 4 patients presented with radial nerve injuries. Of these 4 patients, 1 was injured during the initial trauma, while the remaining 3 were injured due to prior debridement (Table 1).

### Treatments

**Fig. 1:**
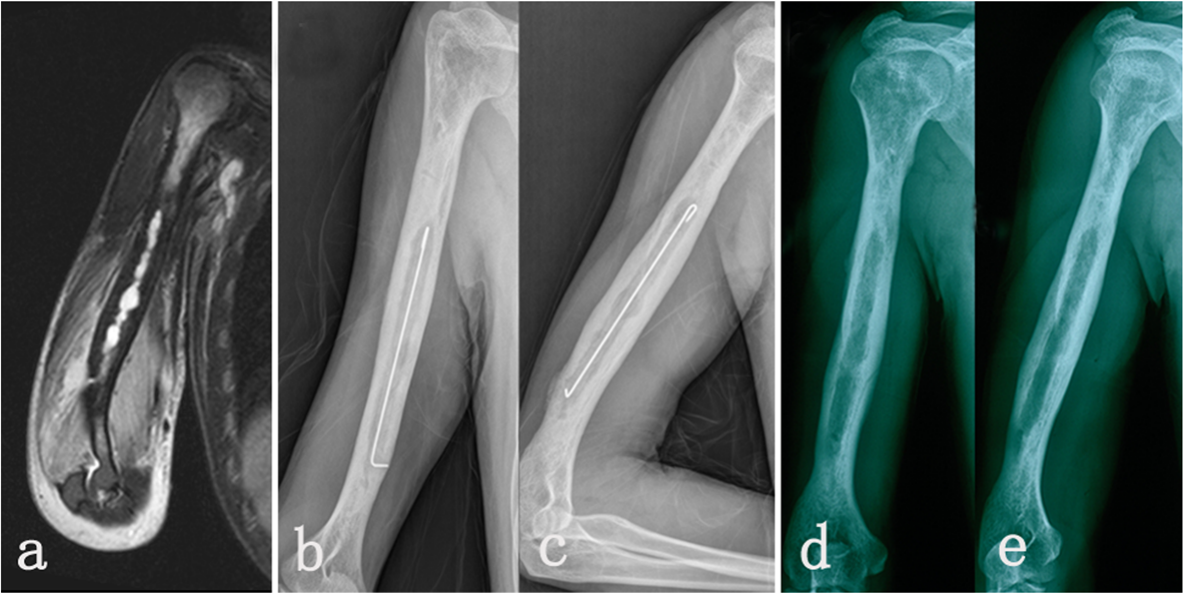
Imaging results from a 23-year-old male with type I humeral osteomyelitis (Patient 4). **a** Preoperative magnetic resonance imaging of the medullary infection in the humerus. **b**-**c** Positive and lateral X-ray after debridement showing medullary management with an antibiotic-coated cement rod. **d**-**e** Positive and lateral X-ray after removing the cement rod in the second stage

**Fig. 2:**
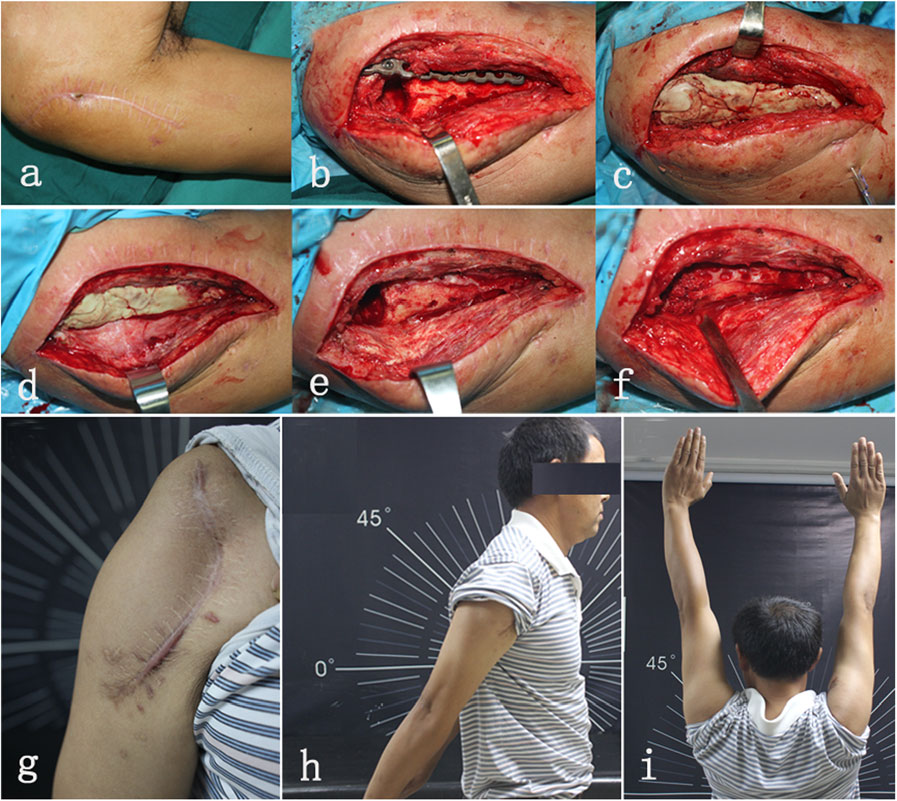
Intraoperative view and functional result from a 44-year-old male with type III humeral osteomyelitis (Patient 13). **a** One sinus tract in the wound in the proximal humerus. **b** Plate fixation after debridement. **c** The antibiotic-coated cement rod filling a bone defect and a coated plate. **d**–**f** Spacer and bone graft were removed in the second stage. **g**–**i** Functional result at the two-year follow-up showing good wound healing progress (**g**), full extension (**h**) and full elevation (**i**)

**Fig. 3:**
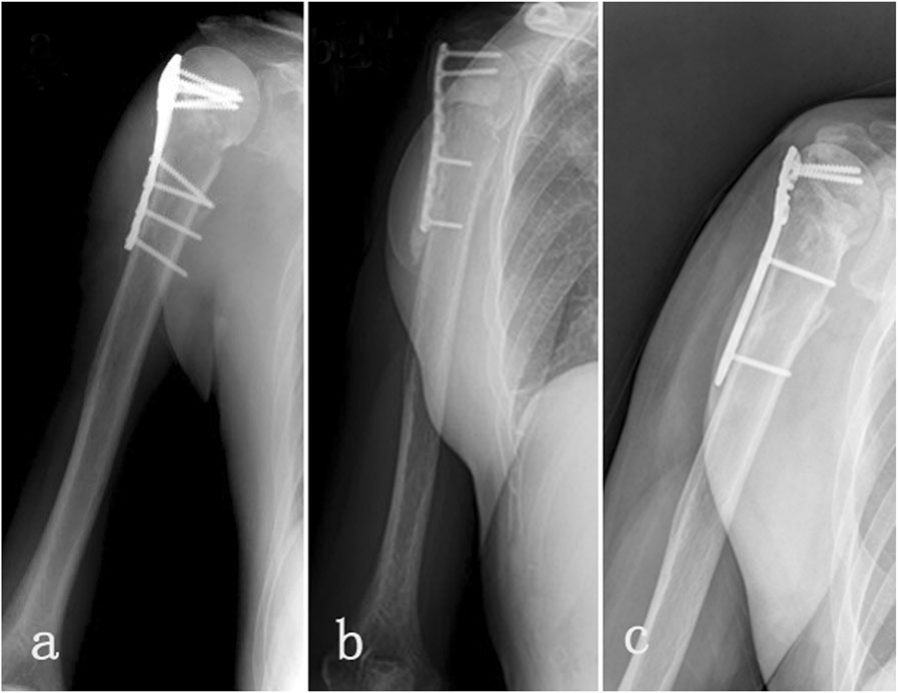
Imaging results of a patient with humeral osteomyelitis (Patient 13) **a** X-ray of a patient with post-traumatic osteomyelitis of the proximal humerus. **b** Post-debridement X-ray with an antibiotic-coated cement spacer and a coated plate for internal fixation. **c** Bone healing state 2 years after bone grafting

**Fig. 4:**
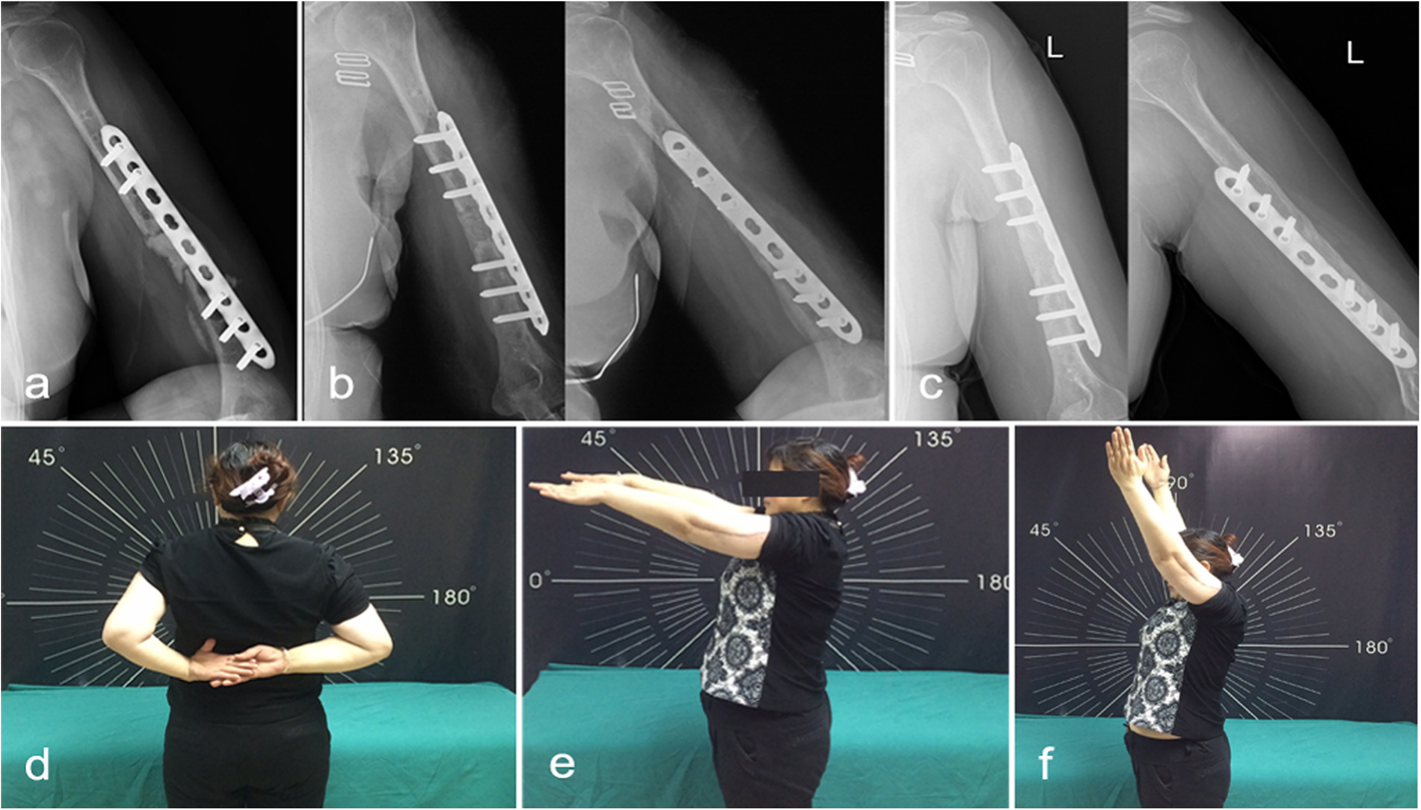
Treatment and follow-up results from a 45-year-old female with type humeral osteomyelitis (Patient 22). **a** AP radiograph shows a 2.8-cm defect after debridement with an antibiotic-coated cement and plate fixation in the first stage. **b** X-ray follow-up 4 months after bone grafting with clinical bone healing. **c** X-ray follow-up after 2 years with bone consolidation. **d**–**f**. Functional result at the final follow-up with good rotation (**d**), good flexion (**e**-**f**)

All patients underwent staged management involving debridement, long-term anti-infective therapy and delayed bone grafting when necessary. In the first stage, 8 patients (type IA) had debridement mainly through intramedullary reaming followed by the introduction of an antibiotic-impregnated cement rod (Fig. 1). Eleven patients with localized cases (type IIIB) were treated with fenestration of the cortical bone on the involved locations (Figs. 2 and 3). Nine patients with diffused cases (type IVB) underwent surgery with resection of the whole osteomyelitis area (leaving 0.8–2.6 cm of segmental defects) (Fig. 4). For these 20 patients, the dead space was filled with an antibiotic-impregnated cement spacer, and 17 patients received a cement-coated plate for fixation after debridement (Fig. 2b and Fig. 4a). In the second stage, the bone defects were small in 16 patients, so they did not require bone grafting when removing the spacer, and 12 patients received a Masquelet bone graft.

Systemic antibiotics were administered for a mean of 4 weeks; they were administered intravenously for 2 weeks and orally for 2 weeks after debridement [6]. The local antibiotics were administered for a mean of 13 weeks (range 9–26) through antibiotic-impregnated cement spacers or rods.

### Follow-ups

Of the 28 patients, 3 patients (MRSA = 2, *S*. *aureus* = 1) showed recurrent infection after the initial debridement procedure. All 3 of these infection cases were haematogenous and had medullary involvement (type IA), 2 of which manifested drainage before second staged surgery, and the other case displayed septic biopsy during the surgery. These patients underwent a second revision, and all of them achieved infection-free bone healing in the final follow-up (Table 1). No severe treatment complications were found. The final average DASH score was 18.14 ± 5.39, while 6 patients with severe comorbidities (olecranarthritis or radial nerve injury) due to prior trauma and surgery had the lowest levels of limb function (mean DASH score was 69.28) and were unemployed. The remaining 2 older patients (age > 60) had retired, and the other 20 patients had returned to work (detailed data are shown in Additional file 2).

### Prognostic factors investigations

We analysed the factors related to the recurrent revision and patients’ functional outcomes (DASH scores). We discovered that the recurrent revision and final DASH score were not related to patient sex, age, infection duration, aetiology, staged bone graft or antibiotic cement-coated plate for internal fixation (*p* > 0.05) (Table 2). The comorbidities severely affected patients’ functional outcomes (*p* = 0.000) (Table 2). In addition, patients with medullary involvement (type I; significant difference between type I versus type III and type IV patients, *p* < 0.05) were more likely to sustain recurrence (Table 2).

**Table 2:** Influencing factors of recurrent revision and functional outcome

Group (*n*)	Recurrent revision *N* (%)	*P* value	Final DASH score *M* ± SD/*M* (range)	*P* value
Total (28)	3 (10.7%)		18.14 ± 5.39	
Gender				
Male (16)	2 (12.5%)		20.80 ± 8.38	
Female (12)	1 (8.3%)	1.000	14.62 ± 6.07	0.579
Age				
< 40 years (16)	3 (18.8%)		25.21 ± 8.51	
≥ 40 years (12)	0 (0.0%)	0.238	8.71 ± 4.53	0.101
Infection duration				
< 12 months (14)	0 (0.0%)		18.27 ± 7.21	
≥ 12 months (14)	3 (21.4%)	0.222	17.62 ± 8.29	0.925
Comorbidity				
Yes (6)	1 (16.7%)		69.28 ± 5.63	
None (22)	2 (9.1%)	0.530	4.20 ± 1.50	0.000
Aetiology				
Post-traumatic (12)	0 (0.0%)		24.23 ± 9.41	
Haematogenous (16)	3 (18.8%)	0.238	13.58 ± 6.29	0.337
Classification				
Type I (8)	3 (33.3%)		1.1 (0-55.6)	
Type III (11)	0 (0.0%)		8.05 (0-77.8)	
Type IV (9)	0 (0.0%)	0.015	5 (0-86.7)	0.359
Bone graft				
Stage graft (13)	0 (0.0%)		15.70 ± 7.56	
None (15)	3 (20.0%)	0.226	20.27 ± 7.83	0.681
Post-debridement fixation				
Antibiotic cement-coated plate (17)	0 (0.0%)	0.050	22.34 ± 7.77	
None (11)	3 (27.3%)		11.67 ± 6.59	0.343

*DASH* disabilities of the arm, shoulder and hand, *mean ± SD* mean ± standard deviation, *M (range)* median (range)

## Discussion

Humeral osteomyelitis is a significant problem for treating surgeons, and the number of publications on humeral osteomyelitis is limited [8, 9, 11, 13]. The aims of this study were to present our experiences in treating patients and determine the factors associated with the outcomes of these specific patients. Previously, the literature reported that the humerus is affected in 2.6–13.3% of all osteomyelitis cases [6], while the incidence based on our database was 3.4%. Moreover, most patients in this series presented with haematogenous forms, which are less common than the post-traumatic forms presented in the literature [3]. In addition, patients may benefit from having a good soft tissue envelope and good blood supply in this area, as these factors make it more difficult to develop an infection after trauma or surgery. Also, *Staphylococcus aureus* was a solitary agent in this series, and most of the patients developed a MRSA infection. To date, the resolution of infections and preservation of functional integrity still remain as clinical challenges [14]. The treatment strategies of humeral osteomyelitis mainly originate from those of osteomyelitis in the lower limbs. When dealing with humeral osteomyelitis, however, certain unique characteristics, including a good soft tissue envelope and good blood supply, as well as complex neuroanatomy (radial nerve), cause the treatment strategies to be slightly different from the general treatment strategies.

Surgical debridement relying on the Cierny-Mader classification and staged approach are general guides for the management of chronic osteomyelitis [5, 12, 15]. According to these principles, we performed debridement, including the removal of all infected dead bone areas, soft tissues and foreign bodies. However, 3 patients with medullary involvement in this series presented persistent infections after debridement. The recurrence was attributed to inadequate debridement in our series, and it seems that medullary infection (type I) is difficult to eliminate in the humerus (*p* < 0.05). This finding is not in accordance with previous findings in the literature, which describe type IV to be the most complex and commonly recurring form in the lower limbs [4, 12]. Undoubtedly, aggressive debridement may increase the odds of infection eradication, but the adjacent radial nerve complicates the treatment, and one should be aware of potential iatrogenic injuries. Three patients (3/28) in our series sustained radial nerve injuries due to prior debridement, which severely affected the patients’ function. Another series reported by Ferreira et al. [6] also showed two patients (2/8) with iatrogenic radial nerve injuries after multiple surgeries. On the other hand, because of the good soft tissue envelope and blood supply in the humerus, we and some authors [12, 16] believe that the general debridement procedure followed by adjunct antibiotic therapy may eventually eradicate infections. Although three patients presented with recurrence and needed a second revision in our series, no patients had iatrogenic nerve injury, and all patients achieved an infection-free state in the final follow-up.

Traditionally, the use of plate fixation is not suitable for osteomyelitis because the implant increases the risk of persistent infections or relapse [9]. Seventeen patients in our series received an antibiotic-impregnated cement-coated plate for internal fixation after debridement. This fixation has previously been demonstrated to be safe and effective for the treatment of infected defects or nonunion in lower limbs [17, 18]. The authors stated that the method can attain a satisfactory level of stability compared with that of traditional external fixation. We first reported the application of this method in the humerus, and it significantly improved patients’ satisfaction and did not improve recurrence in our series (> 0.05). Recently, Pan et al. [19] concluded that the local drug and barrier effect through antibiotic-impregnated cement may reduce subsequent bacterial colonization and the formation of biofilms. Although there are many advantages of this procedure and it is being used more frequently, we suggest that it may be especially suited for patients with better soft tissue envelopes (in the humerus). Moreover, the procedure may solve surgeons’ dilemma in determining whether to retain or remove hardware with regards to the bone healing process and functional integrity.

Another important part of our treatment was the use of local antibiotics in the stage management of humeral osteomyelitis. The methods are widely used in the treatment of the lower extremities, and the antibiotic-loaded bead, spacer and rod are the most commonly used tools for releasing local antibiotics according to the literature [19, 20]. The general staged approach of treating chronic osteomyelitis further allows dead spaces to be treated with antibiotic-loaded spacers (rods). Previously, Ferreira et al. [13] reported eight patients with humeral osteomyelitis (type IV) who had infections cured by the placement of an antibiotic-loaded spacer after debridement. Gallucci et al. [21] also presented a patient (type I) who received an antibiotic-impregnated intramedullary rod and showed no evidence of recurrence at the final follow-up. In our series, 8 patients (type I) were treated with antibiotic-loaded rods and 20 patients (type III or type IV) were treated with a spacer for an average of 13 weeks. We also achieved good results with an 100% infection cure rate at the final follow-up. Care must be taken when the cement becomes exothermic, and actions for the prevention of potential thermal damage are often needed, especially for radial nerves. In addition, the cement may lose its antibiotic activity after several years and increase the risk of relapse and bacterial resistance [20], so the cement needs to be removed completely in the second stage. Generally, we prefer to administer 2 weeks of tailored systemic antibiotics followed by another 2 weeks of oral antibiotics, which was confirmed to be effective in studies in the literature [6].

Some cases in our series performed Masquelet bone grafts in the second stage. This technique [22, 23] was considered to have a biological advantage, in which the induced membrane that forms around the spacer can promote the healing of bone defects. The literature showed many excellent results in the management of lower limb defects [4, 24, 25]. In this series, 13 patients with staged bone grafts all achieved bone union, and the final infection cure of these patients was not different from that of individuals without grafts (> 0.05). In general, reconstruction in the humerus is not a challenge with small bone defects after debridement [10]. The resolution of infections is crucial, and infections need to be carefully assessed if there is a possibility of a residual infection to be present and noted during the second stage. With the consideration of adequate local antibiotics [16] and the biological factors of grafting [22], we often assess the infection status at 8 weeks after debridement. The delayed surgeries in the second stage of this study were mainly delayed for social reasons (an average of 13 weeks). Two patients presented recurrent illnesses in the preoperative assessment, and another patient was confirmed to have a persistent infection by intraoperative biopsy. Previously, Zhang et al. [26] also detected a persistent infection in 22% of the patients with shoulder infection through open biopsy during staged treatment. With the development of quantitative histology [27], we think it will be a positive predictive indicator for sepsis and have an increased role in the prevention of infection relapses.

Osteomyelitis has devastating effects on bone functional integrity and patients’ quality of life [28]. Because of persistent deficits, Liener et al. [14] previously reported that both the psychological well-being and the physical functional capacities of the osteomyelitis population are significantly reduced compared with those of the general population. In this series, although the treatment was able to cure 100% of the infections, some patients still had high DASH scores due to longstanding osteomyelitis and repeated surgeries. The infections and repeated surgeries often result in stiffness of the adjacent joint and increase the risk of nerve injury [13]. Twenty-one patients (75%) had surgeries before this series was conducted, and 6 patients (21%) sustained severe comorbidities as a result (4 cases with radial nerve injury, 2 cases developed traumatic olecranarthritis). These issues severely affected patients’ functional outcomes (*p* < 0.05), and these patients did not return to work. Therefore, patients with humeral osteomyelitis should be referred to an experienced surgeon for professional treatment. This approach will ensure the highest chance of resolving an infection and preserving limb integrity. In addition, this study is limited in that the series was a retrospective cohort study and it did not include a control group. Because few series of humeral osteomyelitis are available in the literature, this paper will add new information for both patients and treating surgeons.

## Conclusion

Humeral chronic osteomyelitis is uncommon but remains a challenging problem for both patients and treating surgeons. The incidence of haematogenous osteomyelitis, caused mainly by *Staphylococcus aureus*, remains very high in the humerus. Appropriate debridement and anti-infective therapy are able to resolve the infection in most patients; however, comorbidities are common and significantly affect patients’ function. Compared with other types of infections, medullary (type I) infections seems to be more challenging to resolve, and the antibiotic-coated cement plate provides favourable fixation without increasing recurrence of infections. Clinicians should be aware of potential iatrogenic nerve injuries when treating these patients with complicated cases, and an experienced surgeon may improve the outcome.

## Supplementary information

**Additional file 1.** Dash-Items.

**Additional file 2: Table S1.** Patients' detail data.

## Data Availability

The datasets used or analysed during the current study are available from the corresponding author upon reasonable request.

## Abbreviations

CRP: C-reactive protein
DASH: Disabilities of the arm, shoulder and hand
ESR: Erythrocyte sedimentation rate
MRSA: Methicillin-resistant *S. aureus*
