# Supplementary material for: Investigating clinical characteristics and prognostic factors in patients with chronic osteomyelitis of humerus
Source: Burns Trauma. 2019 Dec 5;7:34. doi: 10.1186/s41038-019-0173-0 (PMC6894245; doi:10.1186/s41038-019-0173-0)
Supplement: Supplementary file 1 — Additional file 1. Dash-Items. [file 41038_2019_173_MOESM1_ESM.pdf]

DASH-ITEMS

1. Open a tight or new jar
2. Write
3. Turn a key
4. Prepare a meal
5. Push open a heavy door
6. Place an objecton a shelf above your head
7. Do heavy household chores (e.g. wash walls, wash floors)
8. Garden or yard work
9. Make a bed
10. Carry a shopping bag or briefcase
11. Carry a heavy object(over 10 lbs)
12. Change a light-bulb overhead
13. Wash or blow dry your hair
14. Wash your back
15. Puton a pullover sweater
16. Use a knife to cut food
17. Recreational activities which require little effort (e.g.card playing, knitting etc.)
18. Recreational activities in which you take some force or impact through your arm, shoulder or hand (e.g. golf, hammering, tennis etc.)
19. Recreational activities in which you move your arm freely (e.g. playing Frisbee, badminton etc.)
20. Manage transportation needs
21. Sexual activities
22. During the past week, to what extent has your arm,shoulder or hand interfered with your normal social activities with family, friends, neighbours or groups?
23. During the past week, were you limited in your workor other daily activities as a result of your arm, shoulder or hand problem?
24. Arm, shoulder or hand pain 25. Arm, shoulder or hand pain when you perform any specific activity

26. Tingling (pins and needles) in your arm, shoulder or hand
27. Weakness in your arm, shoulder or hand
28. Stiffness in your arm, shoulder or hand
29. During the past week, how much difficulty have you had sleeping because of the pain in your arm, shoulder or hand?
30. I feel less capable, less confidentor less useful because of my arm, shoulder or hand problem
