# Supplementary material for: Investigating clinical characteristics and prognostic factors in patients with chronic osteomyelitis of humerus
Source: Burns Trauma. 2019 Dec 5;7:34. doi: 10.1186/s41038-019-0173-0 (PMC6894245; doi:10.1186/s41038-019-0173-0)
Supplement: Supplementary file 2 — Additional file 2: Table S1. Patients' detail data. [file 41038_2019_173_MOESM2_ESM.pdf]

Full database of the patients studied*

Age (y)	Sex	Classifi-cation	Aetiology	Organisms	Post-debridement fixation	Dead space management	Stage graft	Follow-up time(y)	Outcomes				
									Infection cure	Bone union	Complication	DASH score	Occupation state
29	Male	typeⅠ	Haematogenous	Staph.aureus	none	rod	none	32	cure	union		0	work
22	Male	typeⅠ	Haematogenous	Staph.aureus	none	rod	none	28	cure	union		0	work
21	Male	typeⅠ	Haematogenous	MRSA	none	rod	none	38	cure	union	second revision	0	work
23	Female	typeⅠ	Haematogenous	Staph.aureus	none	rod	none	50	cure	union		2.2	work
61	Male	typeⅠ	Haematogenous	Staph.aureus	none	rod	none	26	cure	union		5	retired
26	Female	typeⅠ	Haematogenous	Staph.aureus	none	rod	none	27	cure	union	radial nerve palsies	55.6	unemployed
51	Female	typeⅠ	Haematogenous	Staph.aureus	none	rod	none	28	cure	union	second revision	0	work
20	Male	typeⅠ	Haematogenous	MRSA	none	rod	none	30	cure	union	second revision	2.2	work
45	Female	TypeIII	Haematogenous	none	none	spacer	none	29	cure	union		7.8	work
72	Female	TypeIII	Posttraumatic	Staphylococcus cohnii urealyticum	ACCP	spacer	autograft	42	cure	union		8.3	retired
25	Female	TypeIII	Posttraumatic	Escherichia coli	ACCP	spacer	autograft	60	cure	union		28	work
34	Male	TypeIII	Haematogenous	none	ACCP	spacer	autograft	36	cure	union		1.1	work
44	Male	TypeIII	Posttraumatic	MRS	ACCP	spacer	autograft	34	cure	union		0	work
20	Male	TypeIII	Haematogenous	MRSA	ACCP	spacer	autograft	24	cure	union		10	work
20	Female	TypeIII	Haematogenous	MRSA	ACCP	spacer	none	26	cure	union		0	work

51	Female	TypeIII	Haematogenous	none	none	spacer	none	50	cure	union	radial nerve palsies	55.6	unemployed
29	Male	TypeIII	Haematogenous	Staph.aureus	ACCP	spacer	none	25	cure	union	traumatic olecranarthritis	77.8	unemployed
19	Female	TypeIII	Haematogenous	Staph.aureus	none	spacer	none	37	cure	union		0	work
50	Male	TypeIII	Haematogenous	none	ACCP	spacer	autograft	26	cure	union		0	work
40	male	TypeIV	Posttraumatic	MRSA	ACCP	spacer	autograft	44	cure	union		6.7	work
54	Female	TypeIV	Posttraumatic	none	ACCP	spacer		62	cure	union		0	work
45	Female	TypeIV	Posttraumatic	none	ACCP	spacer	autograft	42	cure	union		0	work
42	Male	TypeIV	Posttraumatic	tenotrophomonas maltophilia、Escherichia coli	ACCP	spacer	autograft	46	cure	union		3.3	work
20	Male	TypeIV	Posttraumatic	enterobacter cloacae	ACCP	spacer	autograft	34	cure	union		0	work
39	Male	TypeIV	Posttraumatic	none	ACCP	spacer	autograft	25	cure	union	radial nerve palsies	86.7	unemployed
27	Male	TypeIV	Posttraumatic	none	ACCP	spacer	none	27	cure	union	radial nerve palsies	80	unemployed
48	Female	TypeIV	Posttraumatic	none	ACCP	spacer	none	31	cure	union		17.8	work
27	Male	TypeIV	Posttraumatic	MRSA	ACCP	spacer	autograft	33	cure	union	traumatic olecranarthritis	60	unemployed

*MRSA:Methicillin-resistant Staphylococcus aureus;ACCP:Antibiotic cement-coated plate;DASH:Disability of arm shoulder and hand
